# Superior glucose metabolism supports NH_4_^+^ assimilation in wheat to improve ammonium tolerance

**DOI:** 10.3389/fpls.2024.1339105

**Published:** 2024-01-22

**Authors:** Jinling Hu, Qiaomei Zheng, Benjamin Neuhäuser, Chaofeng Dong, Zhongwei Tian, Tingbo Dai

**Affiliations:** ^1^ Key Laboratory of Crop Physiology Ecology and Production Management of Ministry of Agriculture, Nanjing Agricultural University, Nanjing, Jiangsu, China; ^2^ Institute of Crop Science, Nutritional Crop Physiology, University of Hohenheim, Stuttgart, Germany

**Keywords:** ammonium stress, ammonium tolerance, ammonium assimilation, glucose metabolism, wheat

## Abstract

The use of slow-release fertilizers and seed-fertilizers cause localized high-ammonium (NH_4_^+^) environments in agricultural fields, adversely affecting wheat growth and development and delaying its yield. Thus, it is important to investigate the physiological responses of wheat and its tolerance to NH_4_^+^ stress to improve the adaptation of wheat to high NH_4_^+^ environments. In this study, the physiological mechanisms of ammonium tolerance in wheat (*Triticum aestivum*) were investigated in depth by comparative analysis of two cultivars: NH_4_^+^-tolerant Xumai25 and NH_4_^+^-sensitive Yangmai20. Cultivation under hydroponic conditions with high NH_4_^+^ (5 mM NH_4_^+^, AN) and nitrate (5 mM NO_3_^-^, NN), as control, provided insights into the nuanced responses of both cultivars. Compared to Yangmai20, Xumai25 displayed a comparatively lesser sensitivity to NH_4_^+^ stress, as evident by a less pronounced reduction in dry plant biomass and a milder adverse impact on root morphology. Despite similarities in NH_4_^+^ efflux and the expression levels of *TaAMT1.1* and *TaAMT1.2* between the two cultivars, Xumai25 exhibited higher NH_4_^+^ influx, while maintaining a lower free NH_4_^+^ concentration in the roots. Furthermore, Xumai25 showed a more pronounced increase in the levels of free amino acids, including asparagine, glutamine, and aspartate, suggesting a superior NH_4_^+^ assimilation capacity under NH_4_^+^ stress compared to Yangmai20. Additionally, the enhanced transcriptional regulation of vacuolar glucose transporter and glucose metabolism under NH_4_^+^ stress in Xumai25 contributed to an enhanced carbon skeleton supply, particularly of 2-oxoglutarate and pyruvate. Taken together, our results demonstrate that the NH_4_^+^ tolerance of Xumai25 is intricately linked to enhanced glucose metabolism and optimized glucose transport, which contributes to the robust NH_4_^+^ assimilation capacity.

## 1 Introduction

Ammonium (NH_4_^+^) stress is a global challenge that severely affects crop production (Esteban et al., 2016). Accumulation of NH_4_^+^ in soils can be attributed to natural events and human activities, including atmospheric NH_4_^+^ deposition (Liu et al., 2013), soil NH_4_^+^ adsorption (Liu et al., 2008), and localized application of NH_4_^+^‐based fertilizers (Pan et al., 2016; Marino and Moran, 2019). Plants subjected to high NH_4_^+^ conditions display distinct characteristics from those grown in NO_3_^-^ conditions, including external acidification, reduced cationic absorption, imbalances in carbon and nitrogen metabolisms, and oxidative damage (Bittsánszky et al., 2015; Esteban et al., 2016). Over the past two decades, several factors contributing to NH_4_^+^ tolerance have been identified, primarily via studies on NH_4_^+^-tolerant rice (*Oryza sativa*) and *Arabidopsis thaliana*. However, the specific plant traits that are responsible for NH_4_^+^ tolerance, especially in NH_4_^+^-sensitive species such as wheat (*Triticum aestivum*), remain unclear.

To mitigate NH_4_^+^ toxicity, plants must delicately balance NH_4_^+^ uptake, assimilation, and release (Bittsánszky et al., 2015). This balance can be achieved by either regulating transporters to reduce NH_4_^+^ uptake or developing effective detoxification mechanisms to counteract excess NH_4_^+^ accumulation (Ijato et al., 2021). In plants, the high-affinity uptake of NH_4_^+^ is primarily mediated by ammonium transporters (AMTs). Studies showed that *AMT1* gene knockout significantly inhibits NH_4_^+^ uptake, while *AMT1* overexpression enhances NH_4_^+^ permeability in the roots (Ranathunge et al., 2014; Li et al., 2016). In *Arabidopsis*, the three AMT1 proteins (AMT1;1, AMT1;2, and AMT1;3) contribute to approximately 90% of NH_4_^+^ uptake (Yuan et al., 2007). In addition, some studies have pointed to the existence of multiple NH_4_^+^ uptake channels in plants aside from AMT (Esteban et al., 2016), and the simultaneous presence of NH_4_^+^ influx and efflux has been observed in barley (*Hordeum vulgare*) and rice root cells under high external NH_4_^+^ conditions (Britto et al., 2001). Collectively, NH_4_^+^ uptake and efflux by plant roots is complex and needs to be assessed from multiple perspectives. The status of NH_4_^+^ uptake and efflux and the relationship of this NH_4_^+^ movement with NH_4_^+^ tolerance under NH_4_^+^ stress is still unknown.

After NH_4_^+^ is absorbed by plant cells, it is converted into glutamine (Gln) by combining with glutamate. The synthesis of glutamate from 2-oxoglutarate (2-OG) is a critical step in NH_4_^+^ assimilation and cellular defense against NH_4_^+^ stress (Bittsánszky et al., 2015). Under NH_4_^+^ stress, many plant species exhibit an increase in the activities of NH_4_^+^ assimilation enzymes, such as glutamine synthetase (GS, EC 6.3.1.2) and glutamate synthase (GOGAT, EC 1.4.7.1) (Britto and Kronzucker, 2002; Vega-Mas et al., 2019a; González-Moro et al., 2021). Notably, *A. thaliana* mutants lacking the GLN1;2 isoform exhibit excessive NH_4_^+^ accumulation and a high sensitivity to NH_4_^+^ stress (Hachiya et al., 2021), underscoring the significance of this pathway in the protection of plants against NH_4_^+^ toxicity. However, there remains some debate regarding the activity of GS under NH_4_^+^ stress (Jian et al., 2018). Consequently, the variability in the activities of NH_4_^+^ assimilation-related enzymes among NH_4_^+^-tolerant species/cultivars needs to be further investigated.

The principal products of NH_4_^+^ assimilation in plants are nitrogen-rich compounds, primarily amino acids, and proteins. The accumulation of these compounds reflects the capacity of plants to assimilate NH_4_^+^ and adapt to NH_4_^+^ stress (Vega-Mas et al., 2019b). Among the free amino acids, glutamate (Glu), glutamine (Gln), aspartic acid (Asp), and asparagine (Asn) consistently accumulated under NH_4_^+^ stress across various plant species (Jian et al., 2018; de la Peña et al., 2019; Vega-Mas et al., 2019a). Studies suggested that Asn and Gln, as crucial forms of nitrogen storage and transportation, reflect the nitrogen status and regulate NH_4_^+^ uptake and assimilation (Xu et al., 2012; Konishi et al., 2016). Distinctly, a previous study observed that Gln and Asn concentrations in the *Arabidopsis chl1-1* mutant were lower than those in the wild type, indicating that the decline in Gln and Asn may be related to ammonium tolerance in the mutant (Jian et al., 2018). Therefore, different NH_4_^+^-tolerant cultivars might exhibit varied accumulations of NH_4_^+^ assimilates, which might be attributed to varying NH_4_^+^ assimilation capacities, however, it still needs to be validated.

Adequate carbon (C) skeleton supply is also essential to address excess NH_4_^+^ under NH_4_^+^ stress (Britto and Kronzuker, 2005). A classic hypothesis on NH_4_^+^ toxicity suggests that insufficient carbon skeletons in the root lead to NH_4_^+^ poisoning in the plant (Esteban et al., 2016). Numerous studies have reported that excess NH_4_^+^ in the root leads to a reduction in soluble sugar content and enhances the TCA cycle, to produce 2-oxoglutarate and oxaloacetate (OAA) for NH_4_^+^ assimilation (Viktor and Cramer, 2005; Ariz et al., 2013; Vega-Mas et al., 2019a). Conversely, some studies indicated that NH_4_^+^ stress increases soluble sugar content and uncouples carbon and nitrogen metabolism (Jian et al., 2018; Li et al., 2020). The complex relationship between sugar metabolism and carbon skeleton supply under NH_4_^+^ stress is responsible for varying response mechanisms and severities of NH_4_^+^ stress in plants. Improving sugar metabolism and carbon skeleton availability under NH_4_^+^ stress may enhance NH_4_^+^ tolerance in plants.

As a major global crop, wheat is essential to ensure food security for the world’s population. Notably, wheat plants exhibit high sensitivity to NH_4_^+^ stress, especially during the seedling and reproductive stages (Wang et al., 2016; Liu et al., 2021). In recent years, there has been growing evidence that NH_4_^+^ stress adversely impacts wheat seedling growth (Ijato et al., 2021; Liu et al., 2021). However, studies on the precise underlying response mechanisms in different NH_4_^+^-tolerant wheat cultivars are still scarce. In this research, we aimed to investigate the physiological and molecular processes underlying NH_4_^+^ tolerance in wheat plants. We conducted a comparative analysis of NH_4_^+^-tolerant and NH_4_^+^-sensitive cultivars under NH_4_^+^ stress, including growth responses, NH_4_^+^ uptake and assimilation, glucose metabolism, and carbon skeleton supply. The study seeks to test two hypotheses: (i) the NH_4_^+^-tolerant cultivar may have a weaker NH_4_^+^ uptake capacity than the NH_4_^+^-sensitive cultivar, and (ii) the NH_4_^+^-tolerant cultivar may have a stronger sugar metabolism, thus providing more carbon skeletons for NH_4_^+^ assimilation under NH_4_^+^ stress.

## 2 Materials and methods

### 2.1 Plant materials and experimental design

We selected two wheat cultivars (as illustrated in **Supplementary Figure S1**), Xumai25 (NH_4_^+^-tolerant) and Yangmai20 (NH_4_^+^-sensitive), based on the observed tolerance and sensitivity to NH_4_^+^ during pre-experiments (data not shown). The seeds of both cultivars were surface sterilized using a 10% (v/v) H_2_O_2_ solution for 15 min, followed by thorough rinsing with sterile distilled water. Subsequently, the seeds were germinated under dark conditions in Petri dishes until the seed bud was ~1 cm long. Then, the seedlings were transplanted into opaque plastic containers (45 cm × 32 cm × 25 cm, volume: 36 L) filled with water. The seedlings at the two-leaf stage were grown in a modified 50% Hoagland nutrient solution until they reached the four-leaf stage. Following this pre-treatment, the seedlings were divided into two groups. One group was treated with nitrate nitrogen (NN, 5 mM NO_3_^−^-N) nutrient solutions and the other with ammonium nitrogen (AN, 5 mM NH_4_^+^-N) nutrient solution. The concentrations and composition of macronutrients in both treatments are listed in **Supplementary Table S1**. The micronutrient composition in both treatments remained consistent, as previously described by Liu et al. (2021). To ensure a consistent nitrogen supply in each solution, the solutions were refreshed every three days and were continuously aerated to prevent anoxic conditions. The pH of each treatment was adjusted daily to 5.8 using 0.1 mM H_2_SO_4_ or 0.1 mM NaOH. The entire experiment was conducted in a controlled greenhouse environment with a 16 h/8 h light/dark cycle and temperature maintained at 18°C during the day and 8.5°C at night. The light intensity and relative air humidity in the greenhouse were set at 400 µmol m^−2^ s^−1^ and 60%, respectively. We adopted a completely randomized block design, and each experiment was replicated three times. Each replication consisted of three containers, and each container housed 60 plants.

The entire ammonium stress treatment was sustained for 20 days. Seedlings were collected at 0, 1, 3, 5,10, and 20 days after treatment (DAT) to assess biochemical and physiological changes. The leaves, stems, and roots of the seedlings were separated and divided into two segments. One segment was subjected to oven drying at 105°C for 20 min, followed by drying at 85°C, for dry weight and nitrogen concentration measurements. The other segment was promptly frozen in liquid nitrogen and stored at −80°C for subsequent analyses.

### 2.2 Root morphology analysis

After 20 days of treatment, the entire root of each wheat seedling was scanned using a V700 scanner system (Epson, Indonesia). Briefly, eight seedlings per treatment group were randomly selected and labeled before the start of the treatment. The plant roots were rinsed with water, placed in a scanning disk with a small amount of water, laid flat and evenly, and scanned. The obtained root images were analyzed using the WinRhizo Pro V700 1.0 software (Regent Instruments, Canada). The data on the length, volume, surface area, and average diameter of the roots were obtained from the software directly. Additionally, the number of lateral roots was determined by counting directly.

### 2.3 Measurement of root NH_4_^+^ flux

The net NH_4_^+^ influx and efflux at the root surface of two cultivars were determined using Non-invasive Micro-test Technology (NMT Physiolyzer^®^, Younger USA LLC, MA, USA), Xuyue (Beijing) Sci. &Tech. Co., Ltd., Beijing, China, provided the measure services. Wheat seedlings of uniform growth were selected before treatment. The measurement of root NH_4_^+^ influx according to Sun et al. (2022). The seedlings were treated with 5.0 mM NH_4_^+^ solution (mentioned above), and tested directly after 0.17, 2, 6, 24, 72, and 120 hours treatment with the high concentration NH_4_^+^ measuring solution (2.5 mM (NH_4_)_2_SO_4_, 0.1 mM CaCl_2_, pH 5.8), respectively. The measurement of root NH_4_^+^ efflux according to Di et al. (2021), wheat seedlings were treated for 0.5, 6, 24, 72, and 120 hours with the 5.0 mM NH_4_^+^ solution in advance, respectively, and then moved to a low concentration NH_4_^+^ measuring solution (0.1 mM (NH_4_)_2_SO_4_, 0.1 mM CaCl_2_, pH 5.8) for testing. Briefly, two roots were randomly selected from each plant, rinsed with distilled water, and immersed at the bottom of the Petri dish containing fresh measure solution (for the NH_4_^+^ efflux measurement, the roots were equilibrated in measure solution for 20 min). The NH_4_^+^ flux microsensor was positioned at an apex of 1600 μm on the root surface (the position with the maximum net fluxes of NH_4_^+^ selected from our preliminary experiment). Stable data was recorded for 3 min, with 8 replicates for each set of assays.

### 2.4 NH_4_^+^ concentration

The determination of NH_4_^+^ concentration followed the procedure outlined by Balkos et al. (2009). Root samples were collected and subsequently desorbed in a 10 mM CaSO_4_ solution for 5 min to remove extracellular NH_4_^+^. Then the roots were ground to powder in liquid nitrogen, and 0.2 g of the powder was homogenized in 2 ml of pre-cooled 10 mM formic acid. The resulting mixture was subjected to centrifugation at 53,000 × g for 5 min at 2°C. The supernatant was then filtered through a 0.45 μm filter into a 2 mL polypropylene tube and assayed for NH_4_^+^ concentration using the o-phthalaldehyde (OPA) method.

### 2.5 Nitrogen accumulation and amino acid concentration

Fresh root, stem, and leaf samples were freeze-dried and then ground into powder for the following measurements.

For N concentration analyses, approximately 0.1g of the powder was accurately weighed and mixed with 5 ml of H_2_SO_4_. The resulting mixture was heated to 200°C until achieving a clear solution. Subsequently, the reaction was terminated by adding H_2_O_2_. The resulting solutions were then analyzed using ICP-OES (Optima 8000, Perkin Elmer). Plant nitrogen accumulation = (plant dry weight - plant dry weight before treatment) × N concentration.

The total free amino acid was determined using the ninhydrin method, following a previously described protocol with slight modifications (Yokoyama and Hiramatsu, 2003). Briefly, 0.1 g root sample powder was weighed and mixed with the extraction buffer, which consisted of acetic acid/sodium acetate (pH 5.4). Then, centrifuging the mixture and collecting the supernatant. The OD value of the supernatant was measured at 580 nm and recorded using a Pharmacia Ultra Spec Pro UV/VIS spectrophotometer (Pharmacia, Cambridge, England). The final concentration of free amino acid was calculated according to the measured simultaneously with leucine as substrate.

To determine glutamate, glutamine, aspartic acid, and asparagine concentrations, 0.1 g of the root sample powder was weighed and extracted with 3% sulfosalicylic acid (w/v) for 12 hours. Afterward, the mixture was centrifuged at 10,000 g for 10 min, and the resulting supernatant was collected. This extraction process was repeated twice, and all the supernatants were combined and then filtered through a 0.22-μm aqueous film filter. The amino acids in the filtrate were quantified using the Hitachi L-8900 automatic amino acid analyzer (L-8900; Hitachi Corp., Tokyo, Japan), following the method described by Ma et al. (2017).

### 2.6 Soluble sugars and carbon skeleton concentration

The sucrose and fructose concentrations were determined using the resorcinol method described by Zeng et al. (2014). Briefly, 0.1g of the root sample powder (fresh samples were freeze-dried and ground) was weighed and extracted with a sugar extraction solution. For sucrose determination, the supernatant was mixed with 2 M NaOH and incubated at 95°C for 10 min. Subsequently, 0.1 M resorcinol and 10 M HCl were added to the mixture, further incubating at 80°C for 60 min. The concentration of fructose was determined similarly to that of sucrose but without NaOH treatment before the color reaction. Both absorbances were measured at 500 nm using a Pharmacia Ultra Spec Pro UV/VIS spectrophotometer (Pharmacia, Cambridge, England). The concentration of fructose derived from hydrolyzed sucrose was subtracted to determine the free fructose concentration. The concentrations of sucrose and fructose were determined based on the corresponding standard curves.

For the quantification of glucose, pyruvate, 2-oxoglutarate (2-OG), and oxaloacetate (OAA) concentrations of roots, the HPLC method described by Georgelis et al. (2018) was used with some modifications. Approximately 0.2 g of fresh root samples were ground into a powder using liquid nitrogen and then mixed with 4 ml of the extraction solution (preheated 80% ethanol) for 5 min at 80 °C. Subsequently, the mixtures were centrifuged at 12000 g for 10 min, and the resulting supernatants were collected. After the first collection of supernatants, the pellets were resuspended in 2 ml of 50% ethanol, and the extraction procedure was repeated as described above. The supernatants were collected again, and the pellets were resuspended with 2 ml of dd-water and repeated the extraction procedure. All supernatants were collected and vigorously shaken after mixing with an equal volume of chloroform. After the extraction procedure, the aqueous phase was collected, dried under vacuum, and re-dissolved in 1 ml of 50% acetonitrile (acetonitrile: water =50: 50). Before analysis using the anion-exchange HPLC system, the samples were filtered through a 0.45 μm filter membrane. Sugar compounds were separated on a Sugar-D column (4.6×250 mm, Nacalai Tesque Inc., Japan) using a mobile phase of acetonitrile/water (75: 25, v/v) at a 1.0 ml/min flow rate. The column temperature was 40 °C, and the injection volume was 30 µl. The quantification of each sugar was performed by comparing the peak areas of the samples with those of the standard solutions.

### 2.7 Enzyme activity

The root GS activity was determined using a previously described method (Jiang et al., 2017). Briefly, 0.5 g of frozen root samples were weighed and extracted with 1.2 mL of extraction buffer (1 mmol L^-1^ EDTA, 100 mmol L^-1^ pH 7.6 Tris-HCl, 1 mmol L^-1^ MgCl_2_, and 10 mmol L^-1^ β-mercaptoethanol). This reaction mixture was then incubated at 25°C for 5 min and then transferred to a hydroxylamine hydrochloride bath at 25°C for 15 min. Subsequently, the mixture was subjected to chromatography utilizing FeCl_3_ solution. Then, the mixture was centrifuged at 4000 rpm for 10 min at 25°C. Finally, the optical density of the supernatant at 540 nm was measured using the Pharmacia Ultra Spec Pro UV/VIS spectrophotometer (Pharmacia, Cambridge, England).

The activity of glutamate dehydrogenase (GDH, EC 1.5.1) was determined according to the procedure outlined by Skopelitis et al. (2007). Briefly, for the assay of NADH-GDH activity, 2.6 ml of the reservoir solution (115.4 mmol L^-1^ pH 8.0 Tris-HCl, 23.1 mmol L^-1^ 2-oxoglutarate, 231 mmol L^-1^ NH_4_Cl), 0.1 ml 30 mmol L^-1^ CaCl_2_, 0.1 ml 0.2 mmol L^-1^ NAD(P)H, and 0.1 ml deionized water were pre-added to the test tubes. The reaction was then initiated by adding 0.1 ml of root extract (same as that of GS), and the absorbance value was measured at 340 nm using a Pharmacia Ultra Spec Pro UV/VIS spectrophotometer (Pharmacia, Cambridge, England), and again after 3 min to calculate the difference. Test tubes with distilled water instead of NADH and root extracts were used as blank controls. For the analysis of NAD^+^-GDH activity, 2.6 ml of the reservoir solution (115.4 mmol L^-1^ pH 9.3 Tris-HCl, 115.4 mmol L^-1^ L-glutamate, 30 mmol L^-1^ CaCl_2_), 0.05 ml 30 mmol L^-1^ CaCl_2_, 0.1 ml 30 mmol L^-1^ NAD^+^, and 0.15 ml deionized water were pre-added to the test tubes. Other measurement steps were the same as for NADH-GDH. The activity of GDH was expressed as one unit of enzyme activity in terms of the amount of enzyme required to oxidize or reduce 1 μmol of NADH or NAD^+^ min^-1^ at 30 °C.

The activities of GOGAT, hexokinase (HXK, EC 2.7.1.1), phosphofructokinase activity (PFK, EC 2.7.1.11), pyruvate kinase (PK, EC 2.7.1.40), and phosphoenolpyruvate carboxylase (PEPc, 4.1.1.31) were measured using respective kits (catalog numbers: BC0070, BC1465, BC0745, BC0530, BC0540, and BC2190, respectively) purchased from Beijing Solarbio Science & Technology Co., Ltd. (Beijing, China). All enzyme activities were measured using the spectrophotometer as described previously (Chen et al., 2023). Briefly, 0.05 g of each fresh root sample was weighed and ground into powder using a freezer-mill. The powder was then treated with the respective kit reagents as per the manufacturer’s instructions. Finally, the rate of decrease in the absorbance of each reaction solution was obtained using the spectrophotometer.

### 2.8 RT-PCR

Total RNA from root samples was extracted using TRIzol reagent (Vazyme Bio, China). For cDNA synthesis, the HiScript III Q RT SuperMix (Vazyme Bio, China) was employed following the manufacturer’s instruction, and the cDNA samples were diluted 5× before being subjected to qPCR analysis. Real-time quantitative RT-PCR was carried out using the CFX Connect Real-Time PCR Detection System (Bio-Rad, USA) with ChamQ SYBR qPCR Master Mix (Vazyme Bio, China).

The primer sequences for *TaPFK*, *TaHXK*, and *TaPK* were sourced from Li et al. (2019). The primers for *AMT1s* (*TaAMT1.1* and *TaAMT1.2*) were referred to by Ijato et al. (2021). The primer for *TaAmt2.1* was referred to by Porras Murillo et al. (2023). The primers for tonoplast sugar transporter *TaTST*, tonoplast H^+^/glucose symporter *TaERDL*, and the internal reference genes *ACT* and *ADP* were listed in **Supplementary Table S2**. Relative expression levels were determined using the Livak and Schmittgen (2001) method.

### 2.9 Statistical analysis

The experiment was repeated three times during two years. Statistical analyses were performed using SPSS software version 19 (IBM Corp., Armonk, NY, USA). Analysis of variance (ANOVA) was subsequently carried out, and *post hoc* comparisons of means were performed using Duncan’s test. Graphs and tables were generated using Excel and Origin 2018 software (OriginLab, Northampton, MA, USA).

## 3 Results

### 3.1 Dry weight and root morphology

**Figure 1:**
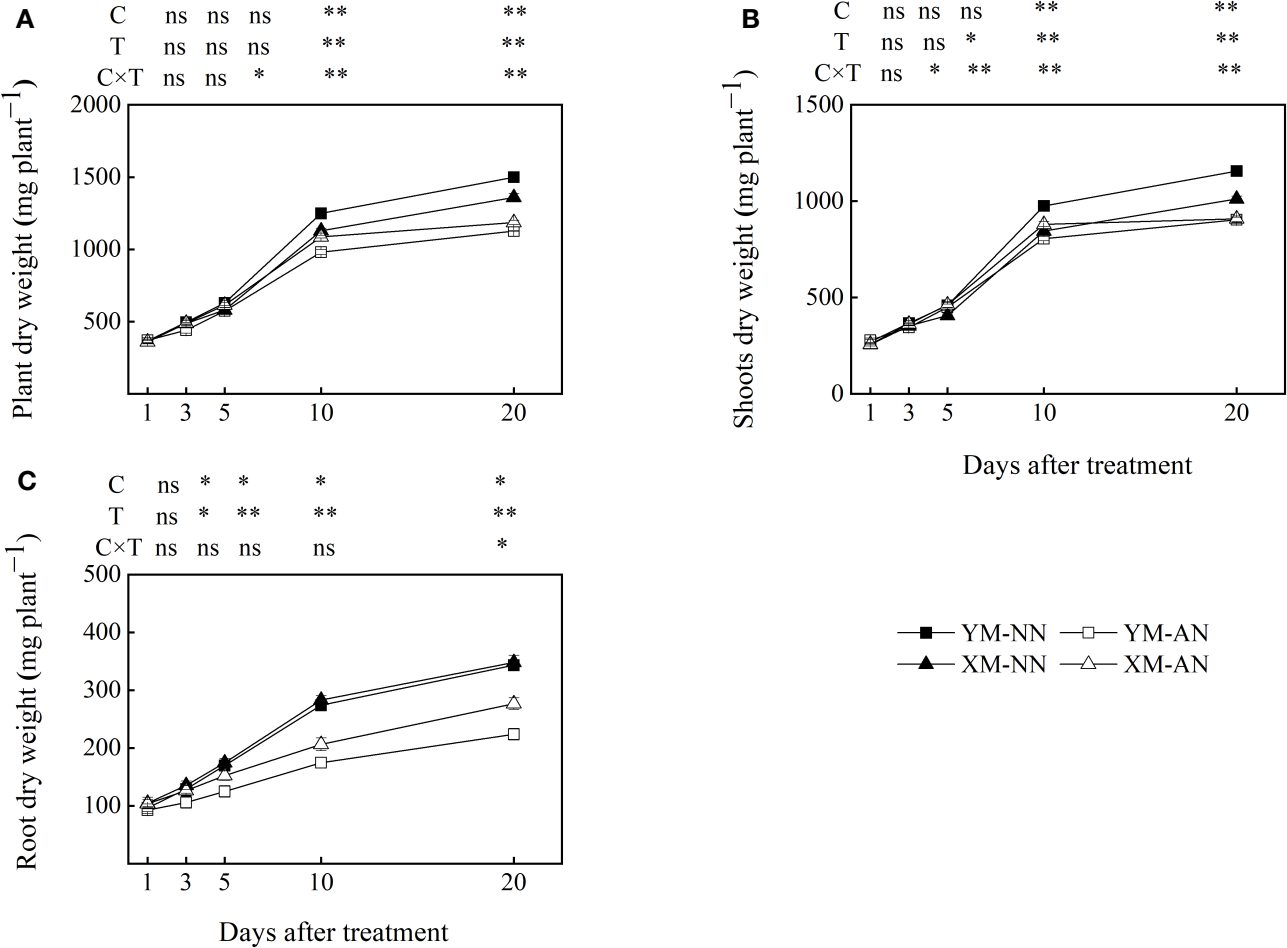
Effect of ammonium stress on biomass accumulation of two different ammonium-sensitive cultivars. **(A)** plant dry weight; **(B)** shoot dry weight; **(C)** root dry weight. Data are given as means of three biological replicates, and error bars indicate SD. NN, nitrate conditions; AN, ammonium stress conditions. YM, NH_4_^+^-sensitive cultivar Yangmai20; XM, NH_4_^+^-tolerant cultivar Xumai25. C, T, and C×T represent the F-value of cultivar, treatment, and the interaction between cultivar and treatment, respectively. The symbols * and ** indicate significant differences at the 0.05 and 0.01 levels, respectively, while ns refers to no significant difference.

We first examined the growth responses of the two wheat cultivars to NH_4_^+^ stress. The AN-treated plants exhibited significantly reduced shoot, root, and total plant dry weight than the NN-treated plants, with the impact being more pronounced in Yangmai20 than in Xumai25 (**Figure 1**). Notably, the decrease in the root dry weight for Yangmai20 commenced at 3 DAT, while that for Xumai25 began at 5 DAT (**Figure 1C**).

Next, we assessed the root morphology of the cultivars to analyze the differential root responses under NH_4_^+^ stress. At 20 DAT, for both cultivars, we observed significantly reduced length, surface area, and volume of both primary and lateral roots for AN-treated plants than NN-treated plants (**Table 1**). After AN treatment, Yangmai20 exhibited more prominent reductions in the length, surface area, and volume of the primary root than Xumai25 (64%, 60%, and 64% vs. 50%, 49%, and 47%, respectively). Similarly, Yangmai20 exhibited more prominent reductions in the length, surface area, and volume of the lateral roots than Xumai25 post-AN treatment (36%, 43%, and 48% vs. 22%, 27%, and 29%, respectively). Moreover, the average diameter of the primary roots of AN-treated plants was comparable to that of NN-treated plants. However, the AN-treated Yangmai20 and Xumai25 exhibited a 23% and 25% increase in the average diameter of the lateral roots and a 33% and 23% reduction in the number of lateral roots, respectively, than their NN-treated counterparts (**Table 1**).

**Table 1:** Effects of ammonium stress on the root morphology of wheat seedlings after 20 days of treatment.

Cultivar	Treatment	Root length (cm)		Root surface area (cm^2^)		Average diameter (mm)		Root volume (cm^3^)		LR numbers
		PR	LR	PR	LR	PR	LR	PR	LR	
Yangmai20	NN	3405 ± 58a	484 ± 12a	332 ± 6.2a	86 ± 2.1a	0.32 ± 0.009a	0.42 ± 0.014c	2.55 ± 0.053a	0.93 ± 0.014b	20 ± 1.0a
	AN	1218 ± 25d	312 ± 10c	132 ± 11.3d	50 ± 1.8c	0.30 ± 0.008a	0.52 ± 0.01a	0.93 ± 0.005c	0.56 ± 0.006d	13.3 ± 0.33c
Xumai25	NN	3037 ± 50b	527 ± 16a	307 ± 6.4b	95 ± 4.6a	0.32 ± 0.011a	0.40 ± 0.019c	2.41 ± 0.109a	1.08 ± 0.015a	23 ± 1.5a
	AN	1528 ± 31c	413 ± 19b	154 ± 6.5c	74 ± 2.2b	0.31 ± 0.014a	0.49 ± 0.009b	1.22 ± 0.033b	0.77 ± 0.023c	17.7 ± 0.23b
*F-value*	*F_Cultivar_*	0.74	23.61**	0.05	25.68**	7.81*	10.8*	1.04	95.07**	48.86**
	*F_Treatment_*	2947.57**	94.23**	902.67**	74**	27.71**	94.54**	362.21**	340.9**	132.25**
	*F_C×T_*	99.43**	3.73	16.04**	5.84	9.62*	0.11	7.99*	2.68	0.46

NN, nitrate conditions; AN, ammonium stress conditions. Yangmai20, NH_4_^+^-sensitive cultivar; Xumai25, NH_4_^+^-tolerant cultivar. PR, primary root; LR, lateral root. Data are means ± standard deviation (SD) of eight wheat seedlings, and different letters indicate significant differences (P<0.05) according to ANOVA. F*_Cultivar_*, F*_Treatment_*, and F*_C×T_* refer to the F-value of cultivar, treatment, and interaction of cultivar by treatment, respectively. * and ** indicate significant differences at the 0.05 and 0.01 levels.

### 3.2 Free NH_4_^+^ concentration

**Figure 2:**
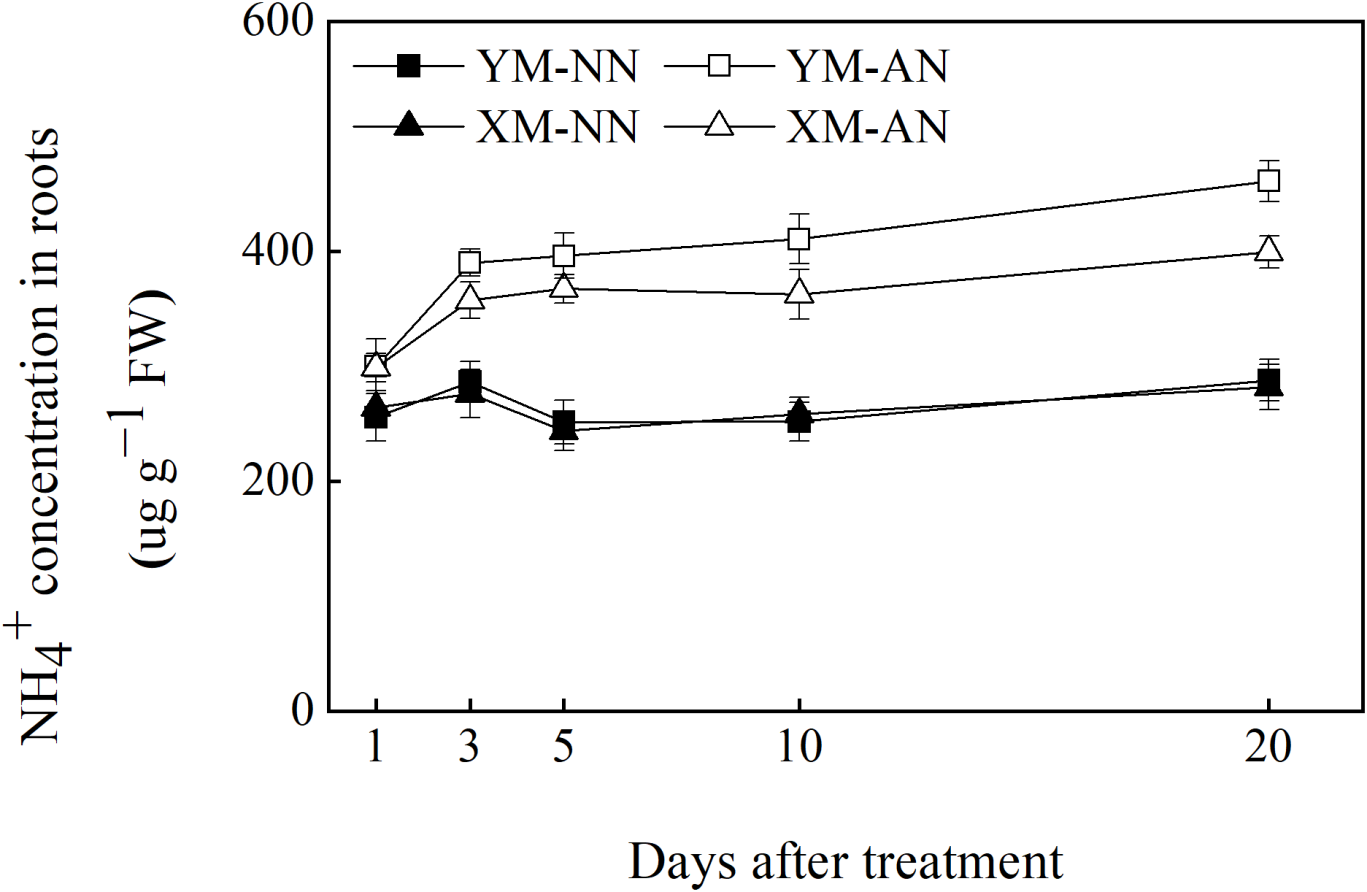
Effects of ammonium stress on root free NH_4_^+^ concentration of wheat seedlings after 1, 3, 5, 10, and 20 days. Data are given as means of three biological replicates, and error bars indicate SD. NN, nitrate conditions; AN, ammonium stress conditions. YM, NH_4_^+^-sensitive cultivar Yangmai20; XM, NH_4_^+^-tolerant cultivar Xumai25.

Accumulation of free NH_4_^+^ in the root contributes to NH_4_^+^ toxicity in plants. Hence, we next compared free NH_4_^+^ accumulation in the roots of the two wheat cultivars. The free NH_4_^+^ concentration in the roots of both cultivars did not differ significantly after NN treatment. However, AN treatment led to an increase in the free NH_4_^+^ concentration of the roots of both cultivars, with a more prominent increase in Yangmai20 than Xumai25 (**Figure 2**).

### 3.3 NH_4_^+^ influx and efflux

**Figure 3:**
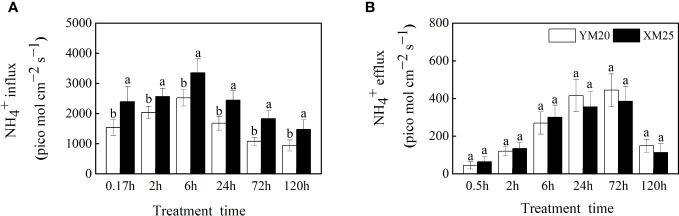
Effects of ammonium stress on root NH_4_^+^ influx and efflux of wheat seedlings. **(A)** NH_4_^+^ influx; **(B)** NH_4_^+^ efflux. Data are given as means of 8 replicates. Error bar labels with different letters indicate significant differences (P < 0.05) between cultivars. YM20, NH_4_^+^-sensitive cultivar Yangmai20; XM25, NH_4_^+^-tolerant cultivar Xumai25.

Changes in influx and efflux of NH_4_^+^ are closely related to NH_4_^+^ concentration in the plant and the severity of NH_4_^+^ toxicity. Here, we employed non-invasive micro-test technology (NMT) to dynamically measure changes in net NH_4_^+^ influx and efflux, thus revealing the differences in root NH_4_^+^ uptake between the two cultivars under NH_4_^+^ stress. The results revealed that treatment with 5 mM NH_4_^+^ stimulated NH_4_^+^ influx in the root of both cultivars, peaking at 6 h after treatment. Notably, NH_4_^+^-tolerant Xumai25 exhibited a more pronounced NH_4_^+^ influx (**Figure 3A**) despite a lower free NH_4_^+^ concentration in the root than NH_4_^+^-sensitive Yangmai20 (**Figure 2**).

NH_4_^+^ efflux was observed in the roots of both cultivars at 0.5 h after treatment, gradually increasing with time and peaking at 72 h after treatment. While Yangmai20 tended to exhibit higher NH_4_^+^ efflux, the efflux did not differ significantly between the two cultivars (**Figure 3B**).

### 3.4 Nitrogen status

**Figure 4:**
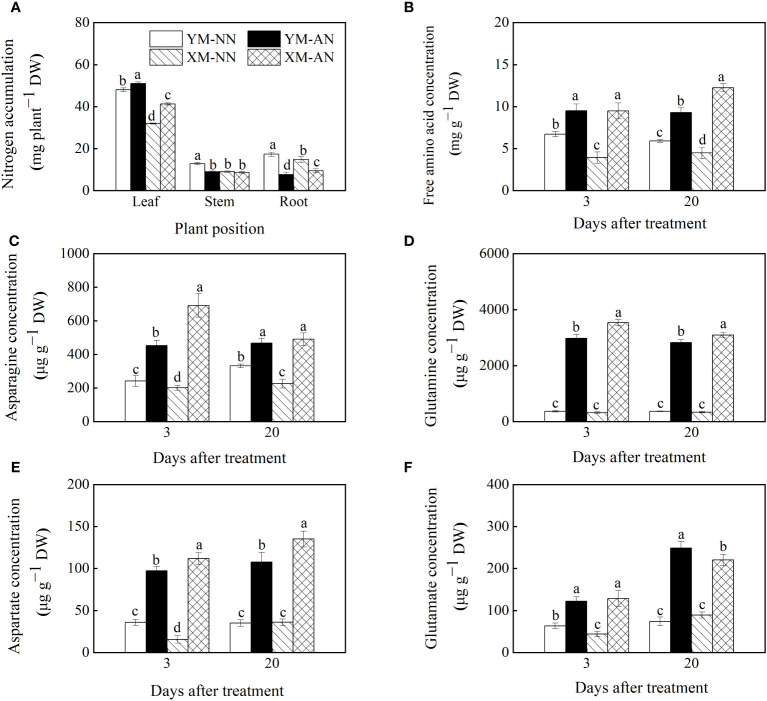
Effects of ammonium stress on nitrogen status of wheat seedlings at 3 and 20 days after treatment. **(A)** Plant nitrogen accumulation; **(B)** Root free amino acid concentration; **(C)** Root asparagine concentration; **(D)** Root glutamine concentration; **(E)** Root aspartate concentration; **(F)** Root glutamate concentration. Data are provided as means of three biological replicates and error bar labels with different letters indicate significant differences (P < 0.05) between cultivars and treatment. NN, nitrate conditions; AN, ammonium stress conditions. YM, NH_4_^+^-sensitive cultivar Yangmai20; XM, NH_4_^+^-tolerant cultivar Xumai25.

Nitrogen accumulation intuitively reflects the plant’s ability to assimilate NH_4_^+^. Hence, we measured and compared the nitrogen status of the two wheat cultivars. Compared to the NN-treated plants, the AN-treated plants exhibited significantly enhanced nitrogen accumulation in the leaves, with more prominent accumulation in Xumai25 than in Yangmai20 (**Figure 4**). Differently, the stem nitrogen accumulation decreased in Yangmai20, while no significant difference was observed in Xumai25. Furthermore, compared to the NN-treated plants, the AN-treated plants exhibited significantly reduced nitrogen accumulation in the roots, with a more prominent reduction in Yangmai20 than Xumai25 (**Figure 4A**).

To elucidate the reasons underlying the varying nitrogen accumulation patterns in the two cultivars, we further measured the concentration of NH_4_^+^ assimilates in the plants. Compared to the NN-treated plants, the AN-treated plants exhibited significantly increased total free amino acid levels in the roots at 3 and 20 DAT, with substantially higher levels in Xumai25 roots than in Yangmai20 roots at 20 DAT (**Figure 4B**). Furthermore, the AN-treated plants exhibited significantly higher Asn, Gln, Asp, and Glu levels than the NN-treated plants (**Figures 4C–F**). Among the AN-treated plants, Xumai25 exhibited a higher increase in Asp, Asn, and Gln levels, but a lower increase in Glu levels than Yangmai20 (**Figures 4C–F**).

### 3.5 Root carbon skeleton supply

**Figure 5:**
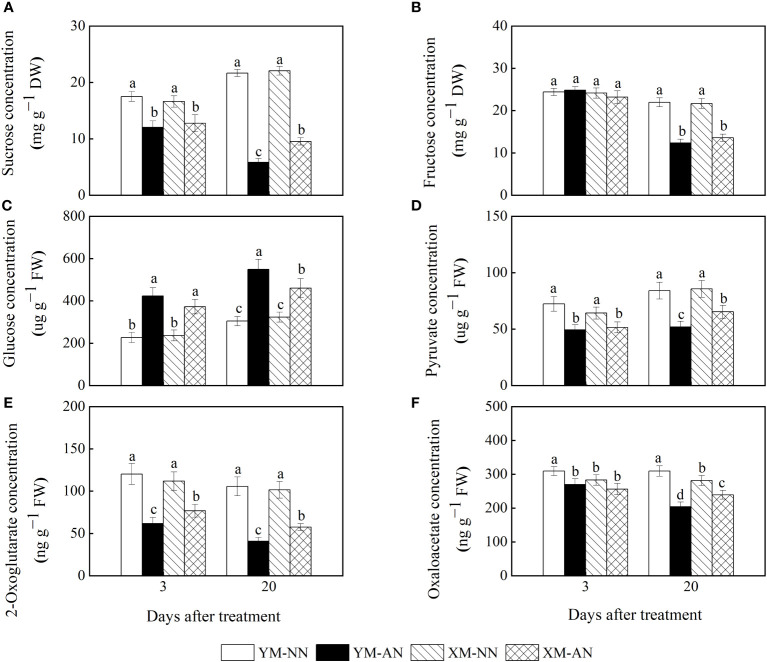
Effects of ammonium stress on root carbon skeleton supply of wheat seedlings at 3 and 20 days after treatment (DAT). **(A)** Sucrose concentration; **(B)** Fructose concentration; **(C)** Glucose concentration; **(D)** Pyruvate concentration; **(E)** 2-Oxoglutarate concentration; **(F)** Oxaloacetate concentration. Data are supplied as means of six biological replicates. Error bar labels with different letters indicate significant differences (P < 0.05) between cultivars and treatment. NN, nitrate conditions; AN, ammonium stress conditions. YM, NH_4_^+^-sensitive cultivar Yangmai20; XM, NH_4_^+^-tolerant cultivar Xumai25.

To investigate the effects of NH_4_^+^ stress on the carbon distribution and supply, we measured the concentrations of sucrose, fructose, glucose, pyruvate, 2-OG acid, and OAA acid in the roots of both wheat cultivars. We observed substantially decreased sucrose levels in the AN-treated plants than the NN-treated plants at 3 and 20 DAT, with higher sucrose levels in Xumai25 than Yangmai20 at 20 DAT (**Figure 5A**). Furthermore, we observed lower fructose concentrations in AN-treated plants than the NN-treated plants at 20 DAT; however, the fructose levels did not differ significantly between the two cultivars at any point in time (**Figure 5B**). Conversely, the glucose concentration steadily increased in both cultivars at 3 and 20 DAT after AN treatment (**Figure 5C**), with 87% and 81% increases in Yangmai20 and 58% and 43% increases in Xumai25, respectively.

Furthermore, the AN-treated plants exhibited significantly reduced pyruvate and 2-OG concentrations than the NN-treated plants (**Figures 5D, E**), with more prominent reductions in Yangmai20 than Xumai25 (**Figures 5D, E**). Similarly, the AN-treated plants exhibited reduced OAA concentrations in the roots than the NN-treated plants at 20 DAT, with more prominent reductions in Yangmai20 than Xumai25 (**Figure 5F**).

### 3.6 Activities of NH_4_^+^-assimilating and sugar-metabolizing enzymes

**Figure 6:**
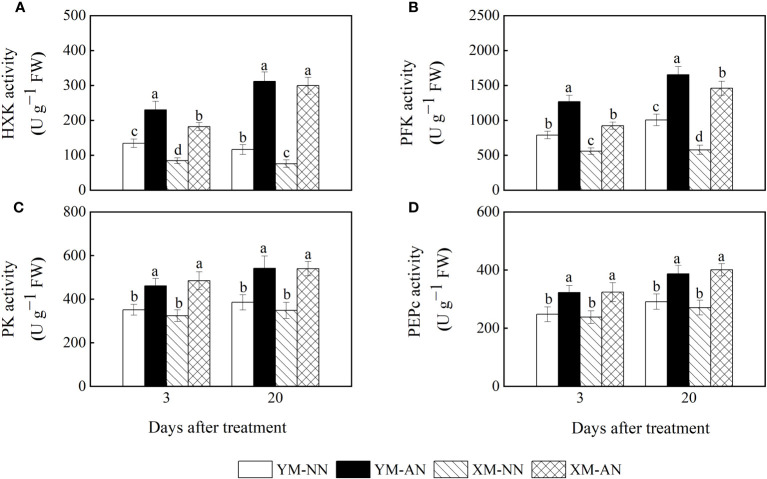
Effects of ammonium stress on the activity of sugar metabolizing enzyme in wheat seedlings at 3 and 20 days after treatment (DAT). **(A)** Hexokinase (HXK) activity; **(B)** Phosphofructokinase (PFK) activity; **(C)** Pyruvate kinase (PK) activity; **(D)** Phosphoenolpyruvate carboxylase (PEPc) activity. Data are expressed as means of three biological replicates. Error bar labels with different letters indicate significant differences (P < 0.05) between cultivars and treatment. NN, nitrate conditions; AN, ammonium stress conditions. YM, NH_4_^+^-sensitive cultivar Yangmai20; XM, NH_4_^+^-tolerant cultivar Xumai25.

To explore the mechanisms underlying lower glucose accumulation in Xumai25, we measured the activities of enzymes related to glucose metabolism. We observed significantly increased activities of HXK, PEPc, PFK, and PK in the AN-treated plants than the NN-treated plants (**Figures 6A–D**). Notably, Xumai25 exhibited a more substantial increase in HXK and PFK activities (294% and 169%, respectively) than Yangmai20 (154% and 64%, respectively) at 20 DAT (**Figures 6A, B**).

**Figure 7:**
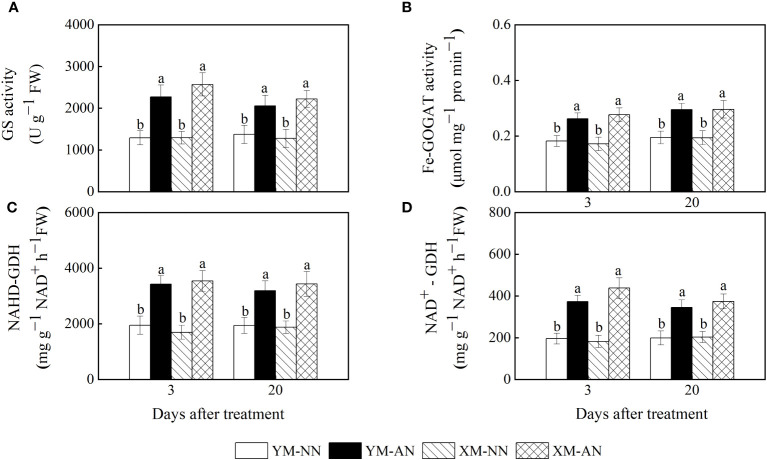
Effects of ammonium stress on the activity of ammonium metalizing in wheat seedlings at 3 and 20 days after treatment (DAT). **(A)** Glutamine synthetase activity; **(B)** Glutamate synthase activity; **(C)** NADH-GDH activity; **(D)** NAD^+^-GDH activity. Data are expressed as means of three biological replicates. Error bar labels with different letters indicate significant differences (P < 0.05) between cultivars and treatment. NN, nitrate conditions; AN, ammonium stress conditions. YM, NH_4_^+^-sensitive cultivar Yangmai20; XM, NH_4_^+^-tolerant cultivar Xumai25.

The activities of NH_4_^+^ assimilation–related enzymes are closely related to the NH_4_^+^ assimilation capacity and NH_4_^+^ tolerance of plants. In this study, the AN-treated plants exhibited higher activities of GS, ferredoxin-dependent glutamate synthase (Fe-GOGAT), NADH-GDH, and NAD^+^-GDH than the NN-treated plants (**Figures 7A–D**). The activities of these enzymes were mildly higher in Xumai25 than in Yangmai20.

### 3.7 Relative gene expression correlates to NH_4_^+^ uptake and carbon supply in roots

**Figure 8:**
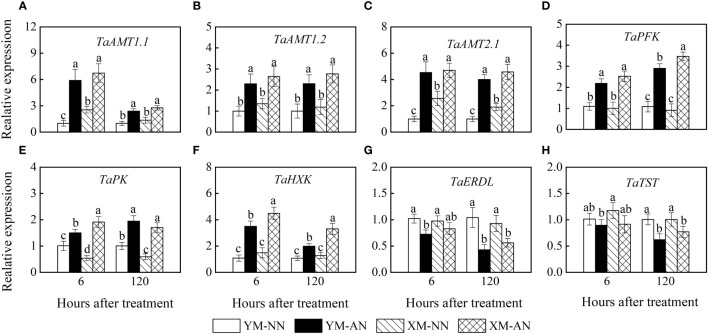
Effects of ammonium stress on relative gene expression in the root of wheat seedlings at 6 and 120 hours after treatment. **(A)** *TaAMT1.1*; **(B)** *TaAMT1.2*; **(C)** *TaAMT2.1*; **(D)** *TaPFK*; **(E)** *TaPK*; **(F)** *TaHXK*; **(G)** *TaERDL*; **(H)** *TaTST*. Data are expressed as means of three biological replicates. Error bar labels with different letters indicate significant differences (P < 0.05) between cultivars. NN, nitrate conditions; AN, ammonium stress conditions. YM, NH_4_^+^-sensitive cultivar Yangmai20; XM, NH_4_^+^-tolerant cultivar Xumai25.

Unlike the NN-treated plants, the AN-treated plants exhibited a rapid upregulation of *TaAMT1.1* and *TaAMT1.2* at 6 and 120 h post-treatment (**Figures 8A–C**), with no significant differences between the two cultivars. Furthermore, the AN-treated plants also exhibited *TaAMT2.1* upregulation in the roots. Moreover, the NN-treated Xumai25 exhibited higher *TaAMT2.1* expression than NN-treated Yangmai20 (**Figure 8C**).

Similarly, we observed upregulation of *TaPK*, *TaHXK*, and *TaPFK* in AN-treated plants at 6 h and 120 h after treatment (**Figures 8D–F**), with more prominent upregulation in Xumai25 than Yangmai25 at 120 h (**Figures 8D–F**).

Tonoplast sugar transporter (TST) and H^+^/glucose symporter (ERDL) mediate glucose transport across the vacuolar membrane. In this study, the AN-treated Yangmai20 exhibited a lower *TaERDL* expression than its NN-treated counterpart at 6 h; however, no significant differences were observed between AN- and NN-treated Xumai25. Moreover, at 120 h, all AN-treated plants exhibited significantly more *TaERDL* downregulation than the NN-treated plants (**Figure 8G**). In addition, the *TaTST* expression did not differ significantly between AN- and NN-treated plants at 6 h; however, this gene was significantly inhibited in the AN-treated plants than the NN-treated plants at 120 h (**Figure 8H**).

## 4 Discussion

It is well known that wheat is sensitive to NH_4_^+^ (Liu and von Wirén, 2017). In the present study, we assessed the responses of two wheat cultivars, NH_4_^+^-sensitive Yangmai20 and NH_4_^+^-tolerant Xumai25, to NH_4_^+^ stress to elucidate the mechanism of NH_4_^+^tolerance in wheat. Our results showed that NH_4_^+^ stress had a significant adverse impact on the growth of wheat seedlings (**Table 1**, **Figure 1**), which is consistent with similar observations in other plant species (Chen et al., 2020; Guo et al., 2021; Tian et al., 2021). Notably, the NH_4_^+^-tolerant cultivar, Xumai25, exhibited a less reduction in root growth and an enhanced NH_4_^+^ accumulation capacity than the NH_4_^+^-sensitive cultivar, Yangmai20, resulting in a superior overall growth of Xumai25 under NH_4_^+^ stress.

### 4.1 Superior root development and stronger NH_4_^+^ uptake enhances NH_4_^+^ tolerance in Xumai25

The phytotoxicity of NH_4_^+^ on root growth, even at moderate concentrations, is a well-known phenomenon across several plant species (Li et al., 2014; Di et al., 2021). The present study showed that NH_4_^+^ stress markedly impacted wheat root morphology and root dry matter accumulation, with a more pronounced effect on the primary root (**Figure 1C**, **Table 1**). This effect was manifested as suppression of root length, surface area, and volume of both cultivars (**Table 1**), aligning with previous observations in *Arabidopsis* (Liu and von Wirén, 2017). The primary root of the NH_4_^+^-sensitive cultivar, Yangmai20, was more substantially affected by NH_4_^+^ stress. Furthermore, lateral roots are known to be highly responsive to nutrient availability (Giehl and von Wiren, 2014). In this study, we observed that the plasticity of lateral roots was adversely affected by NH_4_^+^ stress as evidenced by an increase in root average diameter and a decrease in length, surface area, and volume in both cultivars, with more pronounced effects in Yangmai20 (**Table 1**). Concurrently, NH_4_^+^ stress led to a reduction in the number of lateral roots in both cultivars (**Table 1**), consistent with prior observations in *Arabidopsis* (Li et al., 2013), implying that NH_4_^+^ stress inhibits the germination of lateral roots in wheat (Liu and von Wirén, 2017). Taken together, our findings suggest that 5 mM NH_4_^+^ stress inhibits the growth of primary and lateral roots in the wheat seedlings, resulting in a decrease in root dry matter. The better NH_4_^+^ stress acclimatization capacity of Xumai25, compared with Yangmai20, might contribute to the superior root development in the former.

NH_4_^+^ uptake and transport in plant tissues are predominantly mediated by AMTs. The expression of *AMTs* is influenced by plant species as well as NH_4_^+^ concentration (Li et al., 2017). Previous studies have identified persistent NH_4_^+^ absorption via AMTs as a major contributor to the excessive free NH_4_^+^ accumulation in *Arabidopsis* (Li et al., 2020). Furthermore, exposure to high NH_4_^+^ concentrations tends to suppress the expressions of *AMTs* (Loqué et al., 2006). In the present study, both wheat cultivars exhibited an upregulation of *TaAMT1.1*, *TaAMT1.2*, and *TaAMT2.1* under NH_4_^+^ stress (**Figures 8A–C**), which promoted NH_4_^+^ entry into the root. These findings align with the previous studies on wheat (Li et al., 2017; Ijato et al., 2021), demonstrating that, unlike *Arabidopsis*, wheat did not suppress the expression of *AMTs* under NH_4_^+^ stress to reduce NH_4_^+^ uptake. In addition, the enhanced expression of *TaAMT2.1* in Xumai25 correlated with its superior NH_4_^+^ influx capacity (**Figures 3A**, **8C**).

In addition, previous studies have shown the existence of other NH_4_^+^ uptake pathways in plant roots (Bittsánszky et al., 2015; Esteban et al., 2016). Therefore, to precisely measure NH_4_^+^ influx and efflux from wheat root epidermis, we employed the non-invasive micro-test technology (NMT) (Kühtreiber and Jaffe, 1990), which helps to exclude the influence of other NH_4_^+^ uptake channels on the results. In this study, we observed a higher NH_4_^+^ influx in Xumai25 than in Yangmai20 (**Figure 3A**), consistent with trends observed in ^15^NH_4_^+^ uptake (**Supplementary Figure S2**). Such NH_4_^+^ influx patterns have also been reported among different bamboo species (Zou et al., 2020). In addition, several studies have reported that despite the high net influx of NH_4_^+^ via plant roots, there might be a substantial NH_4_^+^ efflux from the roots to the outside (Britto et al., 2001; Di et al., 2021). Indeed, in the present study, both wheat cultivars exhibited NH_4_^+^ efflux from the root, but there was no significant difference in the amount of NH_4_^+^ efflux between them (**Figure 3B**). Taken together, the higher NH_4_^+^ influx and lower root free NH_4_^+^ concentration in Xumai25 (**Figure 2**), compared with that of Yangmai20, indicates its stronger NH_4_^+^ assimilation capacity, which is related to its higher ammonium tolerance. In addition, these data suggest that the NH_4_^+^-tolerant cultivar has a stronger NH_4_^+^ uptake capacity than the sensitive cultivar, disproving our first research hypothesis.

### 4.2 Superior NH_4_^+^ assimilation capacity positively impacts NH_4_^+^ tolerance in Xumai25

After entering plant cells, NH_4_^+^ is rapidly converted to glutamine and glutamate via the GS-GOGAT-GDH cycle (Xiao et al., 2023). Our study observed a significant increase in the activities of GS/Fe-GOGAT, NADH-GDH, and NAD^+^-GDH in the roots of both wheat cultivars under NH_4_^+^ stress (**Figures 7A–D**), further evidencing the activation of NH_4_^+^ assimilation-associated enzymes in wheat seedlings by NH_4_^+^ stress (Setién et al., 2013; González-Moro et al., 2021). In addition, some studies have suggested that GDH activity is linked to NH_4_^+^ tolerance (Cruz et al., 2006). Indeed, the current study observed a higher NADH-GDH and NAD^+^-GDH activities in Xumai25 than in Yangmai20, indicating a stronger NH_4_^+^ assimilation capacity of Xumai25.

A well-documented strategy for maintaining intracellular NH_4_^+^ levels in various plant species, including wheat, is to enhance NH_4_^+^ assimilation into organic molecules (Setién et al., 2013). In the present study, both cultivars exhibited a significant increase in total free amino acid levels under NH_4_^+^ stress, with Xumai25 having higher free amino acid levels than Yangmai20 (**Figures 2**, **4B**), further evidencing the superior NH_4_^+^ assimilation capacity of Xumai25. A previous study suggested that the metabolic adaptation to NH_4_^+^ in different species is associated with their preference for synthesizing amino acid (González-Moro et al., 2021). In line with a prior study on wheat (Vega-Mas et al., 2019b), the current study observed a substantial accumulation of Asn and Gln in the root under NH_4_^+^ stress (**Figures 4B, C**), highlighting Asn and Gln as major storage amino acids in wheat plants. Additionally, the higher concentration of Asn in the roots of Xumai 25, compared to Yangmai 20, indicates the potential role of Asn in reducing NH_4_^+^ accumulation as well as the better adaptation of Xumai25 to NH_4_^+^ stress (**Figure 4C**).

Contrary to the findings in tomatoes, where NH_4_^+^ stress did not significantly alter Glu concentration (Xun et al., 2020), our study revealed a significant increase in Glu concentration in both wheat cultivars under NH_4_^+^ stress (**Figure 4F**), aligning with other studies on wheat (Setién et al., 2013; Vega-Mas et al., 2019a). Notably, in this present study, the Glu accumulation was lower in Xumai25 than in Yangmai20 (**Figure 4F**), indicating that Xumai25 was able to convert Glu to other amino acids or nitrogenous compounds more efficiently. This efficiency might also contribute to the higher tolerance of Xumai25 to NH_4_^+^ stress (Wang et al., 2020).

### 4.3 More efficient glucose metabolism and transport contribute to the stronger NH_4_^+^ assimilation in Xumai25

NH_4_^+^ assimilation is closely dependent on large amounts of pyruvate entering the tricarboxylic acid cycle to meet the high demand for carbon skeletons for ammonium detoxification (Vega-Mas et al., 2019b). In plants, sucrose is transported from photosynthetic leaves to the roots via the phloem and then hydrolyzed to hexoses (Glc and Fru) (Zhu et al., 2021), followed by further catabolism to provide a carbon skeleton for NH_4_^+^ assimilation. Under NH_4_^+^ stress, the sucrose and fructose levels substantially declined in the roots of both cultivars (**Figures 5A, B**), with a more prominent decline in Yangmai20 roots. According to Cruz et al. (2006), this decline is associated with the depletion of the carbon skeleton by root NH_4_^+^ assimilation. On the other hand, we speculate that it is also related to the inhibition of photosynthesis, which was reported to vary between the two cultivars in our previous study (Hu et al., 2024). In addition, we observed a remarkable increase in glucose concentration under NH_4_^+^ stress (**Figure 5C**), aligning with similar observations in *Arabidopsis* (Jian et al., 2018; Li et al., 2020). This result implies that NH_4_^+^ stress induces glucose accumulation in the root, and the sugar supply status from the shoot is independent of NH_4_^+^ toxicity, in agreement with Jian et al. (2018). Moreover, we observed that Xumai25 exhibited lower glucose accumulation (**Figure 5C**) but higher levels of pyruvate, 2-OG, and OAA at 20 DAT (**Figures 5C–F**) compared to Yangmai20, indicating a superior glucose metabolism in Xumai25.

In plants, glucose is metabolized to pyruvate via glycolysis (Li et al., 2019), and hexokinase, pyruvate kinase, and phosphofructokinase are key enzymes that regulate the process. A study on the transcriptome of duckweed indicated that genes associated with glycolysis are upregulated under NH_4_^+^ stress, thereby regulating carbon metabolism for ammonium detoxification (Tian et al., 2021). Consistently, our results showed that the activities of key glycolysis-related enzymes, such as HXK, PK, and PFK, were significantly increased under NH_4_^+^ stress in both cultivars (**Figures 6A, B**). Moreover, we observed an upregulation of the genes encoding these enzymes (**Figures 8D, F**), further suggesting that glycolysis is enhanced under NH_4_^+^ stress. Importantly, Xumai25 exhibited higher HXK and PFK activities and expression of genes encoding these enzymes than Yangmai20, demonstrating the former has a superior glycolytic capacity. This higher glycolytic capacity of Xumai25 can generate more pyruvate compared to Yangmai20, which explains its lower glucose accumulation and superior NH_4_^+^ assimilation.

Additionally, sugar transport into vacuoles, a crucial aspect of sugar homeostasis, is predominantly facilitated by various classes of sugar transporters in the tonoplast (Zhu et al., 2021). This phenomenon includes H^+^/sugar antiporters (TST) and H^+^/sugar symporters (ERDL), responsible for sugar influx into and efflux from vacuoles, respectively (Klemens et al., 2014). Notably, our study observed that both *TaTST* and *TaERDL* were down-regulated under NH_4_^+^ stress, with the down-regulation being more pronounced in Yangmai20 (**Figures 8G, H**). These results imply that NH_4_^+^ stress inhibits glucose transport, which is associated with glucose accumulation, and Xumai25 had a stronger glucose transport capacity than Yangmai20. Given that NH_4_^+^ stress encompasses osmotic stress (Bittsánszky et al., 2015; Esteban et al., 2016), it is reasonable to hypothesize that the inhibition of glucose transport under NH_4_^+^ stress might be a plant response mechanism aimed at maintaining cellular osmotic potential and mitigating oxidative stress (Xu et al., 2012). Further research is needed to decipher the molecular mechanisms underlying the role of glucose transport under NH_4_^+^ stress.

## 5 Conclusion

**Figure 9:**
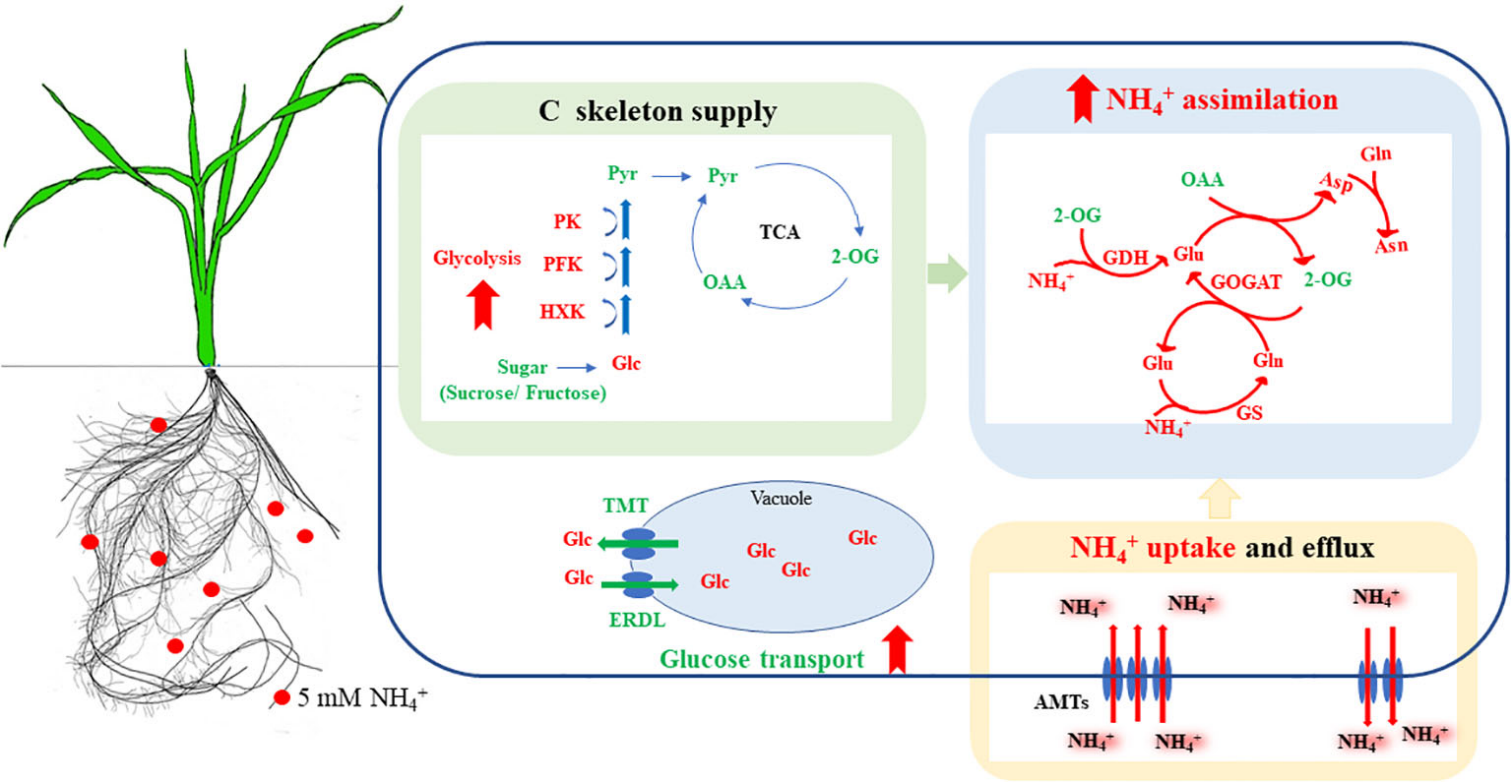
Physiological mechanisms of the enhanced NH_4_^+^ assimilation in NH_4_^+^-tolerant wheat cultivar under NH_4_^+^ stress. Under NH_4_^+^ stress, the NH_4_^+^-tolerant cultivar has an increased NH_4_^+^ uptake, its superior glucose metabolism and transport capacity contributed to the acquisition of more C skeletons, which improved NH_4_^+^ assimilation and reduced the accumulation of free NH_4_^+^ in the root, thus effectively alleviating the inhibitory effects of NH_4_^+^ stress. Red and green, respectively, indicate inhibition/reduction, and activation/increase under NH_4_^+^ stress. Solid red arrows indicate enhanced metabolism of the NH_4_^+^-tolerant cultivar, compared to the NH_4_^+^-sensitive cultivar. AMTs, ammonium transporters; Asn, asparagine; Asp, aspartate; ERDL, tonoplast H+/glucose symporter; GDH, glutamate dehydrogenase; GS, glutamine synthetase; GOGAT, glutamate synthase; Gln, glutamine; Glu, glutamate; HXK, hexokinase; OAA, oxaloacetate; PEPc, phosphoenolpyruvate carboxylase; PFK, phosphofructokinase; PK, pyruvate kinase; TST, tonoplast sugar transporter; 2-OG, 2-oxoglutarate.

In conclusion, our investigation highlights the substantial impact of NH_4_^+^ stress on root growth, NH_4_^+^ uptake and assimilation, and glucose metabolism in different NH_4_^+^ tolerant wheat cultivars. The growth of both wheat cultivars was significantly inhibited under NH_4_^+^ stress. The NH_4_^+^-tolerant cultivar, Xumai25, showed a more robust glucose metabolism and enhanced glucose transport, which provided more carbon skeleton for NH_4_^+^ assimilation and reduced the accumulation of free NH_4_^+^ in the root, thereby exhibiting a stronger NH_4_^+^ assimilation capacity and a better root growth performance (**Figure 9**). This study uncovers the relationship between glucose metabolism, carbon skeleton supply induced by NH_4_^+^ stress, and NH_4_^+^ tolerance of wheat, and will provide a basis for the cultivation and breeding of new NH_4_^+^-tolerant cultivar.

## Data availability statement

The raw data supporting the conclusions of this article will be made available by the authors, without undue reservation.

## Author contributions

JH: Data curation, Formal analysis, Methodology, Writing – original draft. QZ: Writing – review & editing. BN: Writing – review & editing. CD: Writing – review & editing. ZT: Writing – review & editing. TD: Supervision, Writing – review & editing.
