# Supplementary material for: Superior glucose metabolism supports NH4 + assimilation in wheat to improve ammonium tolerance
Source: Front Plant Sci. 2024 Jan 22;15:1339105. doi: 10.3389/fpls.2024.1339105 (PMC10839024; doi:10.3389/fpls.2024.1339105)
Supplement: Supplementary file 1 [file DataSheet_1.docx]

Supplementary Material

**Superior Glucose Metabolism Supports NH_4_^+^ Assimilation in Wheat to Improve Ammonium Tolerance**

Jinling Hu, Qiaomei Zheng, Benjamin Neuhäuser, Chaofeng Dong, Zhongwei Tian, Tingbo Dai*

*** Correspondence:** Tingbo Dai: [tingbod@njau.edu.cn](mailto:tingbod@njau.edu.cn)

## Figures

**
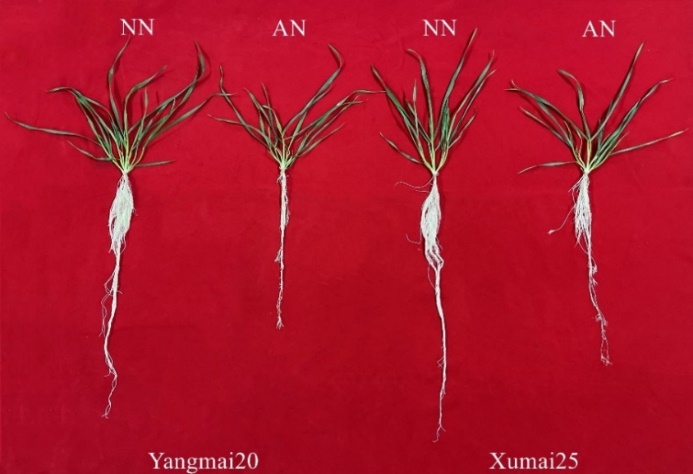
**

**Figure 1.** Comparison of cultivars under NN and AN condition after 10 days. NN, nitrate conditions; AN, ammonium stress conditions. Yangmai20, NH_4_^+^-sensitive cultivar; Xumai25, NH_4_^+^-tolerant cultivar.

## Supplementary Tables

| Treatment | N (5 mM) | K (3 mM) | Ca (1.5 mM) | Mg (1 mM) | P (1 mM) |
| --- | --- | --- | --- | --- | --- |
| AN | (NH_4_)_2_SO_4_ | KH_2_PO_4_ / K_2_SO_4_ | CaCl_2_ / CaSO_4_ | MgSO_4_ | KH_2_PO_4_ |
| NN | Ca(NO_3_)_2_ / KNO_3_ | KH_2_PO_4_ / KNO_3_ | Ca(NO_3_)_2_ | MgSO_4_ | KH_2_PO_4_ |

**Table S1.** The concentration and components of macronutrients in both treatments

NN, nitrate conditions; AN, ammonium stress conditions.

**Table S2**. The primer sequences

| Gene name | Primer sequence (5’ → 3’) | Primer sequence (3’ → 5’) |
| --- | --- | --- |
| *TaTST* | CTGCTTCTTCGTCATGGGGT | TGATGTCGCCGATCCAGAAG |
| *TaERDL* | TCCTAACATCATCGGCTGGC | TAGGATATGACACCGACCCC |
| *ACT* | CAGCAACTGGGATGATATGG | ATTTCGCTTTCAGCAGTGGT |
| *ADP* | GAGATGCGGATCCTGATGGT | CCCCGAGCTTGAGCTTGTAG |

## Figures

**Figure 2.** The NH_4_^+^ uptake of two wheat cultivars after 3 days treatment. Error bars labels with different letters indicate significant differences (P < 0.05) between cultivars. Yangmai20, NH_4_^+^-sensitive cultivar; Xumai25, NH_4_^+^-tolerant cultivar.

Note: 15 NH_4_^+^ measurement method: After 3 days of ammonium stress treatment, the seedlings were cleaned and placed in 5 mM 15NH4SO4 for 10 minutes, followed by harvesting after washing with water and CaSO4. The 15N was measured using a gas isotope mass spectrometer (GIMS).
